# Overall Neutrophil-to-Lymphocyte Ratio and SUV_max_ of Nodal Metastases Predict Outcome in Head and Neck Cancer Before Chemoradiation

**DOI:** 10.3389/fonc.2021.679287

**Published:** 2021-10-08

**Authors:** Jonas Werner, Klaus Strobel, Dirk Lehnick, Gunesh P. Rajan

**Affiliations:** ^1^ Department of Otorhinolaryngology - Head and Neck Surgery, Cantonal Hospital Lucerne, Lucerne, Switzerland; ^2^ Department of Radiology and Nuclear Medicine, Cantonal Hospital Lucerne, Lucerne, Switzerland; ^3^ Department of Health Sciences and Medicine, Biostatistics & Methodology, University of Lucerne, Lucerne, Switzerland; ^4^ Otolaryngology, Head & Neck Surgery, Medical School, University of Western Australia, Perth, WA, Australia

**Keywords:** head and neck cancer, prognosis, biomarkers, tumor metabolism, immune response, radiotherapy resistance, FDG-PET/CT, neutrophil-to-lymphocyte ratio

## Abstract

**Introduction:**

This study investigates the pretherapeutic neutrophil-to-lymphocyte ratio (NLR) with markers of tumor metabolism in 18-fluorodeoxyglucose positron emission tomography/computed tomography (FDG-PET/CT) and their potential prognostic value in head and neck cancer patients prior to primary chemoradiation.

**Materials and Methods:**

NLR and metabolic markers of primary tumor and nodal metastases including maximum standardized uptake value (SUV_max_), metabolic tumor volume (MTV), and total lesion glycolysis (TLG) were retrospectively assessed in a consecutive cohort of head and neck squamous cell cancer patients undergoing primary chemoradiation. The main outcome measure was survival.

**Results:**

The study included 90 patients of which 74 had lymph node metastases at diagnosis. Median follow-up time of nodal positive patients (n=74) was 26.5 months (IQR 18–44). The NLR correlated significantly with metabolic markers of the primary tumor (TLG: r_s_=0.47, *P*<0.001; MTV: r_s_=0.40, *P*<0.001; SUV_max_: r_s_=0.34, *P*=0.003), but much less with FDG-PET/CT surrogate markers of metabolic activity in nodal metastases (TLG: r_s_=0.15, *P*=0.19; MTV: r_s_=0.25, *P*=0.034; SUV_max_: r_s_=0.06, *P*=0.63).

For nodal positive cancer patients, multivariate analysis showed that an increased NLR (HR=1.19, 95% CI=1.04-1.37, *P*=0.012) and SUV_max_ of lymph node metastasis (HR=1.09; 95% CI=0.99-1.19; *P*=0.081) are independently predictive of disease-specific survival. High NLR had a negative prognostic value for overall survival (HR=1.16, 95% CI=1.02-1.33, *P*=0.021).

**Conclusion:**

NLR correlates positively with metabolic markers of the primary tumor, suggestive of an unspecific inflammatory response in the host as a possible reflection of increased metabolism of the primary tumor. SUV_max_ of lymph node metastases and the NLR, however, show no correlation and are independently predictive of disease-specific survival. Therefore, their addition could be used to improve survival prediction in nodal positive head and neck cancer patients undergoing primary chemoradiation.

## Introduction

Primary radiotherapy with concurrent chemotherapy represents an established curative treatment option in advanced-stage head and neck squamous cell cancer enabling organ preservation. Optimized treatment equipment and collimation, altered fractionation, and administration of concurrent chemotherapy have markedly improved the efficacy of radiotherapy in the past decades (1). Depending on the tumor site, local control and survival rates after primary chemoradiation are comparable to those of surgical tumor resection, with the benefit of organ preservation and limited tissue defect (2). However, cancer recurrence or persistence after primary radiochemotherapy (PRCT) remains a regular and challenging situation in which salvage tumor resection is generally considered as the only curative treatment option with reduced survival and functional outcomes (3–5). Additionally, patients undergoing salvage surgery have significantly higher complication rates due to impaired wound healing and tissue health after PRCT (6). Therefore, better prediction of a tumor’s response to PRCT before treatment would enable clinicians to adapt strategies in the first line of treatment. Identifying patients at high risk of cancer recurrence or persistence after PRCT would be useful in triaging these patients for trimodal therapy with upfront tumor resection and postoperative adjuvant treatment. This could reduce recurrence rates and help minimize the necessity of salvage tumor surgeries and hence improve these patient’s survival rates and quality of life.

A few clinical factors such as large tumor volume, extensive nodal disease, or gross cartilage or bone infiltration are recognized risk factors for an incomplete treatment response to PRCT (7–9). The search of additional markers for clinicians to base their therapeutic decision on is ongoing. Metabolic tumor markers in 18-fluorodeoxyglucose positron emission tomography (FDG-PET) imaging including maximum standardized uptake value (SUV_max_), metabolic tumor volume (MTV), and total lesion glycolysis (TLG) have been suggested to predict treatment response to chemoradiation (9–13).

Recently, an elevated pretreatment neutrophil-to-lymphocyte ratio (NLR) has shown to be an easily obtainable prognostic marker which may be used to stratify groups of head and neck cancer patients at risk of tumor persistence or recurrence after PRCT (14–16).

Tumor metabolism is thought to impact the tumor microenvironment. In hypoxic tumor areas, the rate of glucose uptake and fermentation of glucose to lactate is increased (17). This metabolic switch is known as the Warburg Effect and is considered to alter the metabolic status of immune and cancer cells (18, 19). Therapeutic response to chemoradiation largely depends on the function of immune cells which is influenced by tumor metabolism (20, 21).

This study for the first time investigates the pretherapeutic NLR with markers of metabolism of the primary tumor and of nodal metastases in FDG-PET/CT and examines their potential prognostic value in head and neck cancer patients prior to primary chemoradiation. Pretherapeutic metabolic tumor markers in FDG-PET/CT may correlate with the NLR of head and neck cancer patients as a surrogate marker of tumor metabolism triggering an unspecific host immune response. Such a general state of increased inflammation may be reflected by an increased NLR which is itself associated with a worse prognosis (14, 15). In contrast to a specific host immune response to cancer, an increased NLR is considered to diminish tumor response to PRCT, possibly due to a lack of specific immune response in the tumor microenvironment. In addition, elevated markers of general inflammation before treatment may increase the risk for unspecific inflammatory effects after PRCT (22, 23). The aim of this study was to evaluate whether the patient’s pretherapeutic NLR in addition to FDG-PET/CT metabolic markers of the primary tumor or of nodal metastases can predict disease-specific and overall survival before PRCT.

## Methods

### Study Population

The conduction of this study was approved by the regional ethics review board *Ethikkommission Nordwest- und Zentralschweiz* (protocol number 2020-00317). All research was performed in full accordance with relevant guidelines and ethical principles, including the 1975 World Medical Association Declaration of Helsinki. All patients gave general consent to the use of their encrypted medical data for ongoing or future research. A retrospective analysis of all patients undergoing primary radiochemotherapy for oral, oropharyngeal, epipharyngeal, hypopharyngeal or laryngeal squamous cell carcinoma between October 1st, 2011, and October 1st, 2019, at Cantonal Hospital Lucerne, Switzerland, was performed. Inclusion criteria comprised available pretherapeutic FDG-PET/CT images, differential blood analysis, histopathologic diagnosis of squamous cell carcinoma, and treatment with curative intention. Patients with other carcinomas, patients not completing a course of irradiation with at least 66 Gray locally, patients undergoing primary surgical treatment, and patients with ongoing infections or other inflammatory diseases were excluded.

All patients were staged according to the *Union Internationale Contre le Cancer* (UICC), TNM staging for head and neck cancer, 7th edition, 2010 (24). The patients underwent full medical history, physical examination, triple endoscopy with tumor biopsy, and FDG-PET/CT imaging. They were then presented and discussed at the institute’s head & neck multidisciplinary tumor board review.

Detailed data on age, gender, tumor subsite, risk factors including smoking and alcohol consumption, and immunohistochemical expression of human papilloma virus (HPV) infection were obtained in addition to metabolic markers from FDG-PET/CT and complete blood count.

Treatment characteristics including local radiation dose, type and number of cycles of concomitant chemotherapy, salvage tumor resection and/or neck dissection, and follow-up time were assessed. Main outcome measures of statistical analysis were overall survival, disease-specific survival, local and regional recurrence-free survival, and distant metastasis-free survival. In addition, a correlation analysis was performed to investigate the relationship between the NLR and markers of tumor metabolism in FDG-PET/CT. Separate analysis of different tumor subsites was not feasible due to the limited number of patients within the study cohort.

### Analysis of Neutrophil-to-Lymphocyte Ratio

All patients underwent a peripheral blood draw through venipuncture before initiation of treatment. The peripheral blood was analyzed through fluorescence flow cytometry on a Sysmex XN modular system (Sysmex, Kobe, Japan) using a separate channel for white blood cell differentiation analysis called WDF. Leukocyte count and classification into the different subpopulations were performed through lysis of all non-leukocytes (Lysercell^®^) in a first step and dyeing of DNA and RNA particles of the lysed leukocytes (Fluorocell^®^) in a second step. A three-dimensional scatter plot according to size, intracellular structure and fluorescence activity was then formed, allowing for a precise differentiation and count of the leukocyte subpopulations lymphocytes, monocytes, eosinophils, and neutrophils. The neutrophil-to-lymphocyte ratio was then calculated by dividing the total number of neutrophils by lymphocytes in each patient.

### FDG-PET/CT Image Acquisition

Patients were injected with a standardized dose of 3.5 MBq 18-fluorodeoxyglucose (FDG) per kilogram body weight after fasting for at least four hours. All patients had a blood glucose level below 7 mmol/l before imaging. The patients were kept warm prior to tracer injection and instructed to remain in a lying or recumbent position for 60 minutes between FDG injection and image acquisition to minimize muscular FDG uptake and FDG accumulation in brown adipose tissue. All patients received an additional diagnostic neck CT with iodinated contrast medium as part of the PET/CT protocol with injection of 80 ml Ultravist 300 (Bayer Schering Pharma, Germany) at a rate of 3 mL/s *via* a cubital vein and slice thickness of 1.25 mm (pitch 0.875). Scan time per bed position was 2 minutes and scan range from the skull to the knees. Images (15 cm axial field-of-view (FOV)/bed position) were reconstructed by using a standard fully 3D iterative algorithm (ordered subset expectation maximization (OSEM): subsets, 28; iterations, 2; recon matrix, 128 × 128). A low dose CT without intravenous contrast was acquired for attenuation correction with 120kV and variable mAs (dose modulation) and 5 mm slice thickness (pitch 0.875). Images were acquired using a Discovery 600 PET/CT system (GE Healthcare, Waukesha, Wisconsin, USA).

### Tumor and Nodal FDG Metabolism

Selected parameters of FDG metabolism including pretherapeutic SUV_max_, TLG, and MTV of the primary tumor and of the metastatic cervical lymph nodes with the highest SUV_max_ were obtained in each patient with clinically positive nodal status. SUV_max_ was calculated automatically using a standard formula [maximum activity in region of interest ÷ (injected dose × body weight)]. MTV was defined as the sum of the volume of voxels with an SUV exceeding a threshold of 42% of the SUV_max_. TLG was defined mathematically as MTV × SUV_mean_. Correct analysis of FDG uptake was ensured through side-by-side reading of the corresponding CT images of the tumor in the axial, coronal, and sagittal plane. Borders of the regions of interest were set by manual adjustment to exclude adjacent physiologic FDG-avid structures. A written report by a dual board-certified nuclear medicine physician and radiologist was additionally available for pretherapeutic FDG-PET/CT images.

### Statistical Analysis

For continuous variables, univariate distribution may be described utilizing descriptive statistics such as median, interquartile range (IQR), or mean and standard deviation (SD). Pairwise correlations have been determined using Spearman’s rank correlation coefficient. Multiple Cox regression models have been used in order to investigate whether certain combinations of parameters are associated with or can, even in a non-causal manner, explain outcomes such as overall or disease-specific survival. Results are expressed as hazard ratios (HR) with corresponding 95% confidence intervals (95% CI). Survival curves were built according to Kaplan-Meier. Receiver operating characteristic (ROC) curves were used to select the best potential cutoff value for the NLR and metabolic markers including SUV_max_ of lymph node metastases to predict measures of survival. A *P*-value lower than 0.05 was considered to indicate statistical significance. Due to the exploratory nature of the study, no adjustments for multiplicity have been applied. Statistical analyses were conducted with STATA (Version 16.0 or later, StataCorp, College Station, Texas, USA).

## Results

### Patient and Tumor Characteristics

The study cohort consists of 90 patients with squamous cell carcinoma of the oral cavity, epipharynx, mesopharynx, hypopharynx, and larynx of which 74 had nodal metastases (**Table 1**). The median age at diagnosis was 63 years (IQR 58-68). There was a male predominance with 75 (83.3%) male and 15 (16.7%) female patients. Six patients (6.7%) had cancer of the oral cavity, seven (7.8%) of the epipharynx, 43 (47.8%) of the mesopharynx, 21 (23.3%) of the hypopharynx, and 13 (14.4%) of the larynx. Clinical nodal status was positive in 74 (82.2%) patients of which 12 (16.2%) were staged as cN1, 30 (40.5%) as cN2a or cN2b, and 32 (43.2%) as cN2c or cN3.

**Table 1:** Patient demographics and clinical characteristics.

Variable	Distribution	All Patients (n=90)	Nodal Positive Patients (n=74)
**Age**			
Years	Median (Q25–75)	63 (58–68)	63 (58–68)
**Gender**			
Male	n (%)	75 (83.3%)	62 (83.8%)
Female	n (%)	15 (16.7%)	12 (16.2%)
**Risk factors**			
Smoking	Yes (%)	72 (80.0%)	60 (81.1%)
Pack Years	No (%)	14 (15.6%)	11 (14.9%)
(Smokers only)	Missing (%)	4 (4.4%)	3 (4.1)
	Median (Q25–75)	45 (30–60)(n=66)	40 (30–60)(n=55)
Alcohol abuse	Yes (%)	31 (34.4%)	25 (33.8%)
	No (%)	53 (58.9%)	44 (59.5%)
	Missing (%)	6 (6.7%)	5 (6.8%)
p16	Positive	31 (34.4%)	30 (40.5%)
	Negative	28 (31.1%)	24 (32.4%)
	n/a	31 (34.4%)	20 (27.0%)
NLR	Median (Q25–75)	3.3 (2.3–4.6)	3.4 (2.3–5.1)
**Metabolic markers**			
SUV_max_ tumor	Median (Q25–75)	13.1 (9.8–17.3)	13.5 (9.8–17.4)
TLG tumor	Median (Q25–75)	57318 (27409–137172) (n=89)	61990 (30298–145616)
MTV tumor (cm^3^)	Median (Q25–75)	7.7 (4.1–13.1) (n=89)	8.4 (4.2–15.2)
SUV_max_ nodal	Median (Q25–75)	9.6 (5.7–14.7) (n=76)	9.9 (5.7–14.7)
TLG nodal	Median (Q25–75)	45626 (13669–83668) (n=76)	45902 (13854–88805)
MTV nodal (cm^3^)	Median (Q25–75)	7.0 (3.9–11.9) (n=76)	7.0 (4.0–12.4)
**Salvage Surgery**	Yes (%)	23 (25.6%)	19 (25.7%)
	No (%)	67 (74.4%)	55 (74.3%)
**T category**			
cT1	n (%)	4 (4.4%)	4 (5.4%)
cT2	n (%)	19 (21.1%)	17 (23.0%)
cT3	n (%)	40 (44.4%)	30 (40.5%)
cT4	n (%)	27 (30.0%)	23 (31.1%)
**N category**			
cN0	n (%)	16 (17.8%)	0 (0.0%)
cN1–cN2a	n (%)	18 (20.0%)	18 (24.3%)
cN2b–cN3	n (%)	56 (62.2%)	56 (75.7%)

NLR, Neutrophil-to-Lymphocyte Ratio; SUV_max_, Maximum Standardized Uptake Value; TLG, Total Lesion Glycolysis; MTV, Mean Tumor Volume.

The median pretherapeutic SUV_max_ of the primary tumor (n=90) in the whole cohort was 13.1 (IQR 9.8–17.3), while the median SUV_max_ of the metastatic lymph node with the highest metabolic activity in each patient (n=74) was 9.9 (IQR 5.7–14.7). Median follow-up time for all nodal positive patients (n=74), on which the survival analyses were based, was 26.5 months (IQR 18–44).

### Treatment Characteristics

All patients (n=90) were treated with either intensity-modulated radiotherapy (IMRT) or volumetric modulated arc therapy (VMAT) with a mean total dose of 70.9 Gray locally (SD 1.28). Eighty-three patients (92.2%) received concomitant chemotherapy of which 60 (66.7%) were based on cis- or seldom carboplatin, while 30 (33.3%) consisted of cetuximab. Patients on average completed 5.4 cycles of concomitant chemotherapy (SD 1.19). Salvage neck dissection was performed in 21 patients (23.3%) after a median time of 6.5 months (IQR 5.5–10) because of recurrent or persistent metastatic lymph nodes after PRCT. Ten patients (11.1%) had to undergo salvage tumor resection due to recurrence or persistence of the primary tumor. Median time to salvage tumor resection was 15.5 months (IQR 12-26). Final pathology confirmed tumor-free resection margins (R0) in all of these patients.

### Correlation Analysis

**Figure 1:**
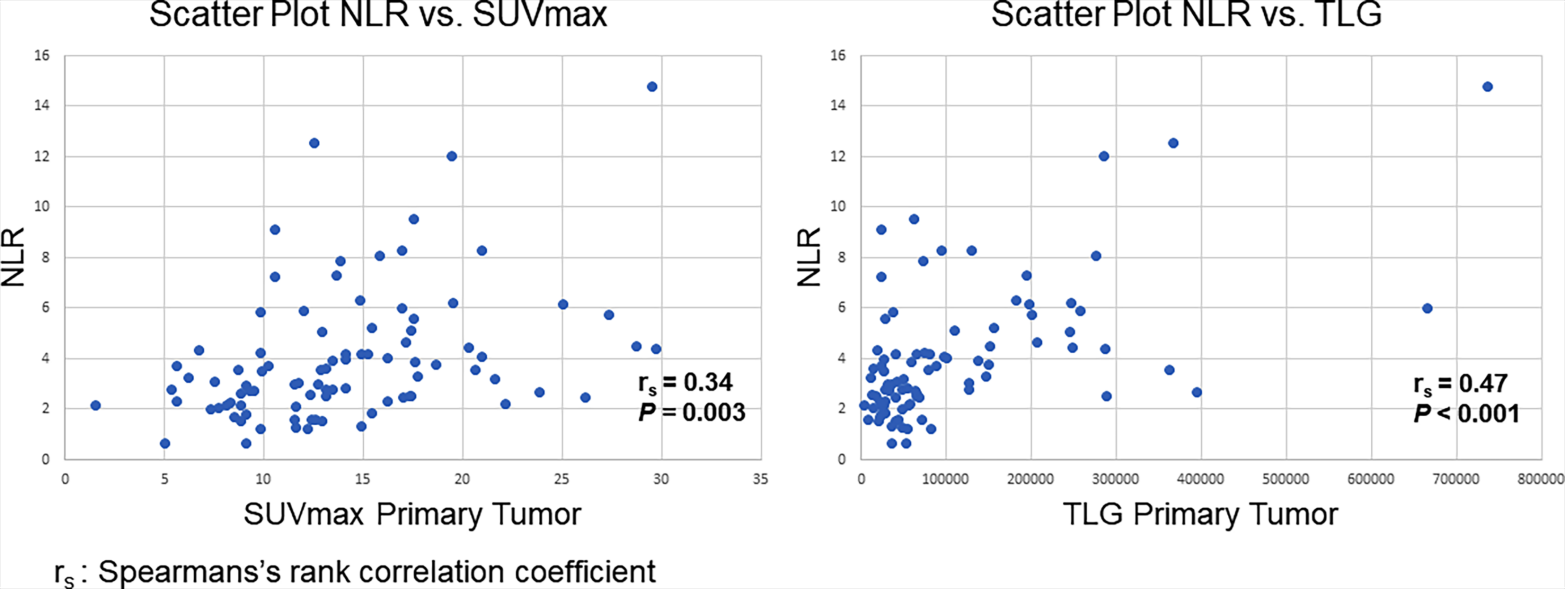
High NLR shows a significant positive correlation with metabolic markers of the primary tumor (SUV_max_: r_s_ = 0.34, *P*=0.003; TLG: r_s_ = 0.47, *P* < 0.001; MTV: r_s_ = 0.41, *P* < 0.001).

Correlation analysis was carried out to evaluate the relationship between the NLR and metabolic markers from FDG-PET/CT of the primary tumor and of the metastatic lymph node with the highest SUV_max_ (**Table 2**). An increased NLR showed a significant positive correlation with the primary tumor’s TLG (r_s_=0.47, *P*<0.001, **Figure 1**), MTV (r_s_=0.40, *P*<0.001), SUV_max_ (r_s_=0.34, *P*=0.003, **Figure 1**), and clinical T-stage (r=0.38, *P*<0.001). The correlation of the NLR with any of the FDG-PET/CT-derived nodal metabolic markers (TLG: r_s_=0.15, *P*=0.19; MTV: r_s_=0.25, *P*=0.034; SUV_max_: r_s_=0.06, *P*=0.63, see **Table 2**) or clinical N-stage (r=-0.08, *P*=0.47) was much less pronounced.

**Table 2:** Correlation analysis.

cN+ Patients (n=74)	*NLR*
**Metabolic Markers**	
SUV_max_ tumor	r**_s_** = 0.34 (*P* = 0.003)
TLG tumor	r**_s_** = 0.47 (*P* < 0.001)
MTV tumor(cm^3^)	r**_s_** = 0.40 (*P* < 0.001)
**SUV_max_ nodal**	r**_s_** = 0.06 (*P* = 0.63)
**TLG nodal**	r**_s_** = 0.15 (*P* = 0.19)
**MTV nodal (cm^3^)**	r**_s_** = 0.25 (*P* = 0.034)

r_s_, Spearman’s rank correlation coefficient.

P, p-value.

### Survival Analysis

Survival analysis was performed for nodal positive patients (n=74) only. Cumulative overall survival (OS) within the follow-up period was 68.9%. Disease-specific survival (DSS) was 77.0% while distant metastasis-free survival (DMFS) was 58.1%. Of the 20 patients developing distant metastasis, 13 patients (65.0%) presented with pulmonary metastases. For those 20 patients, the median time to the diagnosis of distant metastasis was 11 months (IQR 9.5–16).

Thirteen patients (17.6%) demonstrated disease persistence after completing PRCT and 17 (23.0%) had a local or regional cancer recurrence after a median of 12 months (IQR 11–20).

Pretherapeutic patient and cancer related characteristics were assessed in regard to their predictive value for means of survival after PRCT.

For DSS and OS of nodal positive patients at diagnosis, multiple Cox regression models have been fitted, including NLR, TLG, MTV, and SUV_max_ of the primary tumor as well as TLG, MTV, and SUV_max_ of the lymph node metastasis. Utilizing Akaike’s and the Bayesian information criteria (AIC and BIC), the initial model for DSS could be reduced in a stepwise procedure to a model with the NLR and SUV_max_ of the lymph node metastasis. This analysis showed that an increased NLR (HR=1.19, 95% CI=1.04–1.37, P=0.012) and the SUV_max_ of the lymph node metastasis with the highest metabolic activity in each patient (HR=1.09; 95%CI=0.99-1.19; *P*=0.081) were independently predictive of DSS. Metabolic parameters of the primary tumor (SUV_max_, TLG, MTV) did not offer additional predictive value if used in addition to the NLR as they positively correlate with the NLR.

For OS, in a stepwise Cox regression procedure, the original model could be similarly reduced and ended up with a model that retained only the NLR as a remaining factor. An increased NLR had also prognostic value for overall survival (HR=1.17, 95% CI=1.02–1.33, *P*=0.021).

**Figure 2:**
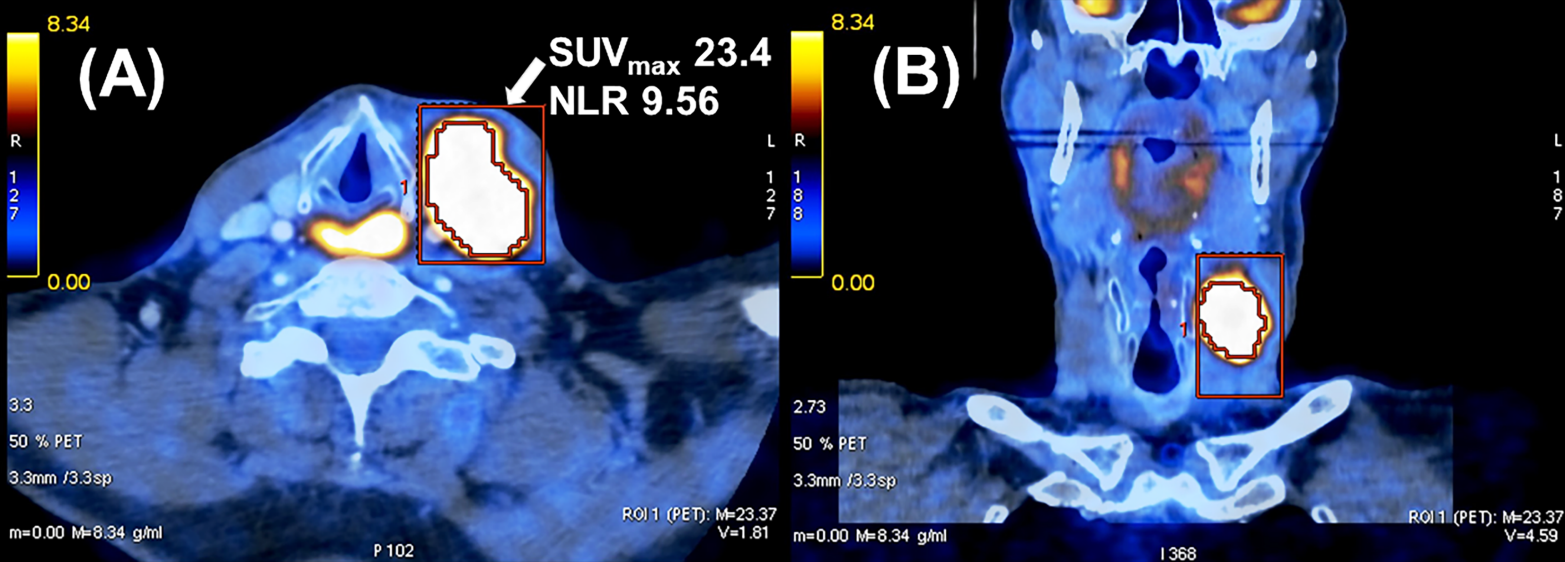
Example of a cT4 cN2c cM0 hypopharyngeal carcinoma in a 62-year-old male patient. He developed locoregional tumor recurrence with distant metastases and died 13 months after completion of radiochemotherapy. **(A)** Axial and **(B)** coronal view of fused PET/CT image of nodal metastasis.

**Figure 2** shows an example of a patient with a high NLR and SUV_max_ of nodal metastasis who developed locoregional recurrence and distant metastases causing his death 13 months after completion of chemoradiation.

**Figure 3:**
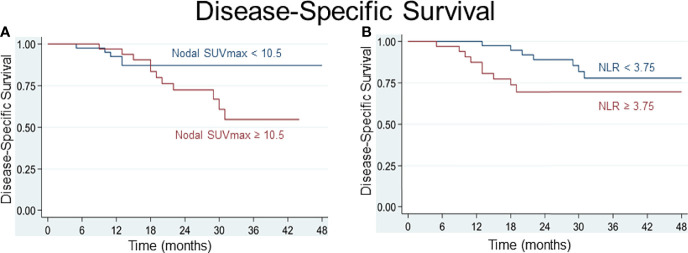
Patients with a pretherapeutic SUV_max_ of nodal metastasis ≥ 10.5 **(A)** and an NLR ≥ 3.75 **(B)** are at risk of worse disease-specific survival.

**Figure 4:**
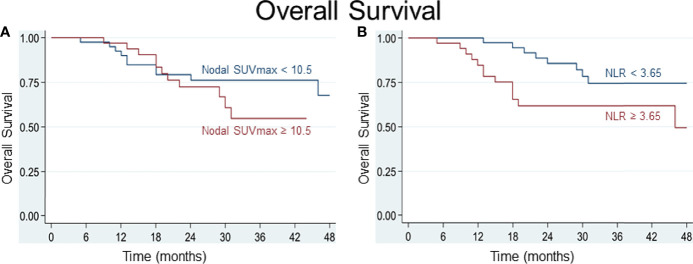
**(A)** A best potential cutoff value of 10.5 was determined for SUV_max_ of nodal metastasis and shows that patients with an SUV_max_ of nodal metastasis ≥ 10.5 are at greater risk of dying. **(B)** An NLR ≥ 3.65 distinguished best between patients at lower (NLR < 3.65) and higher (NLR ≥ 3.65) risk of dying.

Various cutoff values for the NLR and for parameters of FDG uptake were tested using receiver operating characteristic (ROC) curves. For the SUV_max_ of lymph node metastasis, a best potential cutoff value of 10.5 for both DSS (sensitivity 71%, specificity 63%, area under ROC curve (AUC) 0.67) and OS (sensitivity 57%, specificity 61%, AUC 0.59) was determined. For the NLR, a value below or above 3.75 for DSS and 3.65 for OS distinguished best between patients at lower or higher risk of worse survival (for DSS: sensitivity 59%, specificity 61%, AUC 0.60; for OS: sensitivity and specificity 61%, AUC 0.61). Based on the determined cutoff values, time-to-event analyses were performed and Kaplan-Meier curves built. Comparative Kaplan-Meier survival analysis shows that patients with an NLR≥3.65 or ≥3.75, respectively, and a nodal SUV_max_≥10.5 at diagnosis are at risk of worse disease-specific and overall survival (**Figures 3**, **4**).

The limited number of patients in the study cohort did not allow to analyze different tumor subsites separately or subdivide into HPV-positive and HPV-negative patients without risk of beta error (25).

## Discussion

This study investigates the prognostic value of pretherapeutic tumor markers and patient characteristics available at diagnosis with the aim to facilitate clinical decision making between PRCT and upfront surgery with the possibility of adjuvant radiochemotherapy in patients with advanced head and neck squamous cell cancer. In accordance with recent research (10, 12, 14, 15), the prognostic value of both metabolic tumor markers in FDG-PET/CT and of the neutrophil-to-lymphocyte ratio (NLR) could be confirmed in our patient cohort. For the first time, we investigated the relationship between these prognostic markers and demonstrated how the NLR correlates positively with metabolic markers of the primary tumor. Interestingly, the NLR barely correlated with metabolic markers found in nodal metastases of head and neck carcinomas. Hence, the addition of the pretherapeutic NLR and FDG-PET/CT metabolic parameters of nodal metastases could be utilized to better predict treatment response to PRCT. This is exemplified in our patient cohort in which the patient’s NLR and the SUV_max_ of nodal metastases were independently predictive of disease-specific survival in nodal positive cancer patients before PRCT (**Figure 4**). We suggest that the pretherapeutic NLR and SUV_max_ of nodal metastases are best used in addition for prediction of treatment outcome as this increases the predictive power concerning disease-specific survival compared to using each marker individually. Addition of another potential predictor of survival with a significant correlation to the NLR may offer only a limited contribution to improve survival prediction as their predictive power may largely be covered already by the NLR. Combinations of the NLR with the metabolic parameters of the primary tumor did not improve the Cox regression models in terms of model selection criteria. SUV_max_ of lymph node metastases is, according to our data, very weakly correlated with the NLR and therefore provides additional predictive power if used in addition to the NLR.

The statistical analyses performed in this study aimed to identify prognostic factors independent of the TNM-classification in the prediction of survival after PRCT. Therefore, we did not integrate T-stage into our modeling for survival prediction. However, adding clinical T-stage as an explanatory variable to the model further improves prediction. With a HR of about 2 for DSS and 2.5 for OS per increasing unit, T-stage may even become the most influential factor. Nevertheless, even in this case the estimators of the influence of the NLR and SUV_max_ of the lymph node metastasis (or the NLR alone with respect to OS) remain quite robust.

The role of the NLR as prognostic indicator in head and neck cancer was recently supported in a systematic review and meta-analysis including 33 cohorts with over 10’072 patients in which an elevated NLR significantly predicted poorer OS and DSS (14). Seventy-five percent of the included studies determined NLR cutoff values between 2.17 and 3 to best predict OS and 2.50–2.59 for DSS, respectively. In our patient cohort, an NLR cutoff value of 3.75 for DSS and 3.65 for OS seemed to offer the best discrimination (e.g. according to the Liu method or the method identifying the cutpoint on the ROC curve closest to 0 or 1).

An interaction between the local and systemic inflammatory responses within the tumor microenvironment has long been recognized (26). An elevated NLR implies relative neutrophilia and lymphopenia. While high counts of activated neutrophils are known to inhibit cytolytic activity of T-cells and natural killer cells (27), systemic lymphopenia may reflect a decreased presence of lymphocytes in the tumor microenvironment representing a reduced specific host immune response to the tumor (28). Tumor infiltrating lymphocytes are part of the specific host immune response contributing to therapy-mediated tumor control in solid tumors, possibly triggered by the recognition of tumor neoantigens unmasked by the effects of radiochemotherapy (29–31).

While neutrophils are part of the general unspecific immune surveillance, they may promote tumor progression in the tumor microenvironment through local immune suppression. In a process termed as respiratory burst, neutrophils generate reactive oxygen species (ROS) which interfere with lymphocyte functions (32, 33). Neutrophils appear to favor glycolytic metabolism (34). In a tumor microenvironment with limited glucose supply (35), however, a subset of immature neutrophils engages in oxidative mitochondrial metabolism. This eventually leads to accumulation of reactive oxygen species (ROS) which are thought to suppress T cell functions (32).

Tumor-infiltrating neutrophils are often exposed to microenvironments with a low level of oxygen and glucose (34, 36). Activated neutrophils show elevated oxygen consumption during respiratory burst and induce oxygen depletion to promote inflammatory hypoxia in the tumor microenvironment (34, 37).

An increased NLR may reflect neutrophil dominance in the local tumor environment with a subsequent immune suppressing effect of oxidative neutrophils which in turn may diminish the specific host immune tumor response mediated by lymphocytes.

Various studies demonstrate the prognostic value of markers of tumor metabolism in FDG-PET/CT in head and neck cancer (10, 38, 39). These metabolic parameters may be used as surrogate markers of tumor hypoxia which is known to adversely affect tumor response to radiotherapy (40–42). Most previous studies put the emphasis on the predictive value of SUV_max_ (9, 38) and volumetric parameters such as MTV and TLG (10, 43) of the primary tumor. In our patient cohort, however, increased SUV_max_ of the lymph node metastasis with the highest SUV_max_ in each patient showed the best predictive value in addition to the NLR regarding disease-specific survival. We determined a best potential cutoff value for nodal SUV_max_ of 10.5 to be predictive of OS and DSS in our patient cohort. Similarly, Inokuchi et al. describe that an increased nodal SUV_max_ is a significantly unfavorable factor for survival (44). In their patient cohort of 178 nodal positive head and neck squamous cell carcinoma patients, an elevated nodal SUV_max_ provided important information for the selection of patients suitable for planned neck dissection after PRCT. A nodal SUV_max_ cutoff value of 6.0 was determined as best predictor of disease-free survival in their study.

A similar study by Cacicedo et al. (45) in 58 patients with locally advanced head and neck cancer undergoing pretreatment FDG-PET/CT demonstrated that nodal SUV_max_ may be prognostic for distant metastasis-free survival as nodal SUV_max_>5.4 presented an increased risk for distant metastases (HR 3.3; 95% CI 1.17–9.25; *P*=0.023).

SUV_max_, when compared to the volumetric parameters, has the advantage of being a standardized, easily applicable and available marker (38) which does not vary due to spill-over of adjacent FDG-avid structures (46).

We hypothesize that poorly oxygenated tumors show increased FDG uptake and metabolism (47) which may induce an unspecific inflammatory response in the host. The results from our patient cohort analysis support such a relationship in which the pretherapeutic NLR correlates positively with markers of FDG metabolism of the primary tumor. Interestingly, this was not the case for metabolic markers of nodal metastases, as they showed no significant correlation with the NLR. The reason for this paradox remains largely unknown and requires further investigation. One possible explanation may lay in the time gap between the development of the primary tumor and of nodal metastases. The initial transformation of normal cells into cancer cells is considered to trigger an inflammatory response which plays a key role in the development of cancer as many inflammatory signaling pathways are activated in tumorigenesis (48). Inflammation induces tumor cell proliferation through activation of tissue-repair responses following genetic mutations (26, 49). Formation of the primary tumor precedes lymphatic metastasis. The formation of nodal metastases appears to be less associated with systemic inflammation compared to initial tumorigenesis. This may partly be a result of advanced cancer immune evasion over time. Moreover, lymphogenic cancer spread does not seem to trigger an equally strong inflammatory response as observed during primary tumor formation.

The metabolic state of the tumor microenvironment is influenced by heterocellular interactions, oxygen and nutrient availability, as well as oxidative stress (50). Tumor progression affects metabolism and composition of immune stroma, supporting tumor growth and evasion of immune defense responses. As a consequence of oxygen shortage, tumor cells change to anaerobic glycolysis. This metabolic switch has shown to be associated with immune suppression (18).

Furthermore, metabolic shifts fuel various aspects of macrophage activation (51) which may promote local tumor persistence after radiotherapy (52). Rafat et al. (52) demonstrated in an orthotopic breast cancer model that macrophages were recruited to irradiated tissue and led to subsequent accumulation of circulating tumor cells in the absence of CD8+ T-cells. Tumor cell recruitment was mitigated using Maraviroc, a CCR5 (Cysteine-Cysteine Chemokine Receptor 5) receptor antagonist inhibiting macrophage infiltration. This exemplifies how the type of host immune response and its interactions with tumor metabolism may play a role in tumor survival and recurrence after radiotherapy. A better understanding of these interactions potentially offers the possibility of new targeted treatment options to suppress unspecific host immune responses such as inhibitors of macrophage infiltration.

The goal of this study was to assess the value of pretherapeutic biomarkers and patient characteristics in the prediction of tumor response to PRCT by the means of survival analysis. Late effects of PRCT and resulting deficits of organ function such as swallowing disorders were not investigated. The limitations of this study are its retrospective design and the limited number of patients in our cohort which did not allow to perform subanalyses or stratification as per tumor site. The prognostic role of the tumor’s HPV status may have been underrated in our patient cohort due to the relatively low number of patients which increased the likelihood of beta error (25).

Further investigation of pretreatment markers potentially predictive of survival should evaluate different tumor sites separately, taking their distinctive tumor biology and carcinogenesis into account.

**Figure 5:**
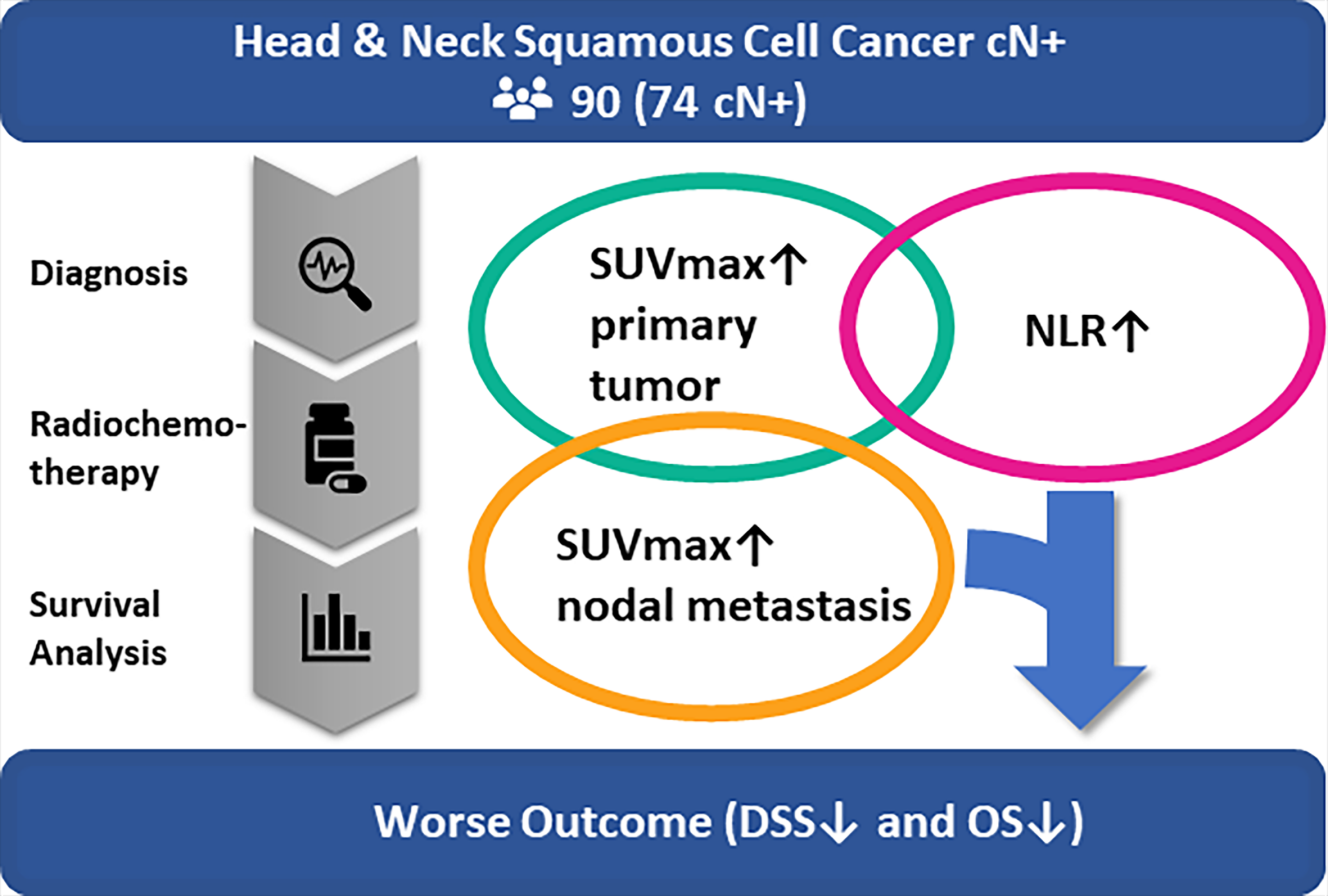
Multivariate analysis of our patient cohort of nodal positive head and neck cancer patients undergoing primary chemoradiation shows that the NLR and SUV_max_ of nodal metastasis are predictive of disease-specific survival and overall survival. There was a significant correlation between NLR and SUV_max_ of the primary tumor but not between NLR and nodal SUV_max_. Therefore, the NLR and SUV_max_ of nodal metastasis could be used in addition to the TNM-classification to improve survival prediction and support treatment decision making in nodal positive head and neck cancer patients.

In conclusion, increased tumor metabolism may induce an unspecific host immune response as metabolic markers of the primary tumor correlate positively with the NLR. SUV_max_ of lymph node metastases and the NLR, however, show no significant correlation and are, added as independent factors in a model, predictive of disease-specific survival (**Figure 5**). Further studies may investigate whether such an addition of biomarkers to the TNM-classification can improve survival prediction prior to PRCT and hence support treatment decision making in nodal positive head and neck cancer patients.

## Author’s Note

This work was presented at the virtually held 2020 Swiss Society for Oto-Rhino-Laryngology Head and Neck Surgery, Annual Meeting, November 12^th^, 2020, Switzerland.

## Data Availability Statement

The original contributions presented in the study are included in the article. Further inquiries can be directed to the corresponding author.

## Ethics Statement

The studies involving human participants were reviewed and approved by *Ethikkommission Nordwest- und Zentralschweiz*. The patients/participants provided their written informed consent to participate in this study.

## Author Contributions

Basic study idea by JW. Patients search and extraction of patient related data by JW. JW and KS extracted the data related to nuclear imaging. JW built the figures and wrote the first draft of the manuscript. DL performed statistical analysis. Manuscript editing and review by GR, KS, and DL. JW, KS, DL, and GR have participated substantially to the study. All authors contributed to the article and approved the submitted version.

## Conflict of Interest

The authors declare that the research was conducted in the absence of any commercial or financial relationships that could be construed as a potential conflict of interest.

## Publisher’s Note

All claims expressed in this article are solely those of the authors and do not necessarily represent those of their affiliated organizations, or those of the publisher, the editors and the reviewers. Any product that may be evaluated in this article, or claim that may be made by its manufacturer, is not guaranteed or endorsed by the publisher.
